# N,S-Co-Doped Carbon Dot-Reinforced Biopolymer Films with Enhanced Barrier, Antioxidant, and Antimicrobial Performance for Active Food Packaging

**DOI:** 10.3390/polym18182215

**Published:** 2026-09-11

**Authors:** Suleiman A. Althawab

**Affiliations:** Department of Food Science & Nutrition, College of Food and Agricultural Sciences, King Saud University, Riyadh 11451, Saudi Arabia; salthawab@ksu.edu.sa

**Keywords:** chitosan/PVA, nanocomposite films, active food packaging, antibacterial activity, antioxidant properties

## Abstract

The development of sustainable active food packaging materials with multifunctional protective capabilities is highly desirable for improving food safety and extending shelf life. In this study, sulfur- and nitrogen-rich carbon dots (GLCDs) were synthesized from glycine and lipoic acid through a facile hydrothermal approach and subsequently incorporated into chitosan/poly(vinyl alcohol) (PVA) matrices to fabricate multifunctional nanocomposite films. Structural and physicochemical characterization suggested the successful incorporation of GLCDs within the polymer network and suggested favorable interactions between the nanofillers and the chitosan/PVA matrix. The GLCD-incorporated films exhibited enhanced mechanical strength, improved thermal stability, reduced water vapor permeability, and superior UV-shielding performance compared with pristine chitosan/PVA films. Furthermore, the nanocomposite films demonstrated significantly enhanced antioxidant activity, achieving approximately 60% DPPH and 90% ABTS radical scavenging efficiency at higher GLCD loading. The films also exhibited strong antibacterial activity and demonstrated preservation potential based on qualitative observations during storage by reducing visible spoilage and maintaining structural integrity. In addition, the developed films showed excellent cytocompatibility toward NIH-3T3 fibroblast cells with high cell viability after 5 days of culture. The synergistic combination of GLCDs and chitosan/PVA matrices provides an effective strategy for developing biodegradable, multifunctional, and cytocompatible active packaging materials with potential food-packaging applications.

## 1. Introduction

The increasing demand for safe, sustainable, and high-performance food packaging materials has accelerated the development of biodegradable polymeric systems capable of extending food shelf life while reducing environmental impact [1]. Conventional petroleum-derived plastic packaging materials, although widely used because of their low cost and favorable mechanical performance, generate persistent environmental pollution and pose serious ecological concerns due to their poor biodegradability and long-term accumulation in ecosystems [2,3,4]. In recent years, bio-based polymeric films derived from naturally abundant materials have emerged as promising alternatives for active food packaging applications owing to their biodegradability, biocompatibility, film-forming capability, and low toxicity [5]. Among them, chitosan has attracted substantial attention because of its inherent antimicrobial activity, excellent biocompatibility, and ability to form transparent films [6,7]. However, pristine chitosan films often suffer from limited mechanical strength, poor moisture resistance, and inadequate long-term stability, restricting their practical application in food preservation systems [8]. To overcome these limitations, blending chitosan with poly(vinyl alcohol) (PVA) has been widely explored to improve flexibility, structural integrity, and film-forming performance through intermolecular hydrogen-bonding interactions [9]. The chitosan/PVA composition used in this study (1.0 g chitosan and 1.5 g PVA) was selected based on previous reports demonstrating that chitosan/PVA blends containing approximately 40–50 wt% chitosan provide a suitable balance of film-forming capability, mechanical performance, flexibility, and barrier properties [10]. In such systems, PVA improves the flexibility and structural stability of chitosan films, while chitosan contributes biodegradability and antimicrobial functionality through strong intermolecular hydrogen-bonding interactions with PVA [11,12]. Recent active-packaging studies have demonstrated the effectiveness of multifunctional systems combining antioxidant and antibacterial properties with food-preservation performance. For example, Gao et al. developed betulin/HPβCD nanofibers with antioxidant, antibacterial, and strawberry-preservation activity, while Zhang et al. reported nerolidol/HPβCD nanofibers with quantitative antibacterial activity and suppression of *Botrytis cinerea* lesion development during strawberry storage [13,14]. These studies highlight the growing interest in multifunctional active-packaging materials and the importance of evaluating both functional properties and preservation performance.

Beyond passive protection, modern food packaging technologies increasingly emphasize the development of active packaging systems capable of preventing microbial contamination, delaying oxidative degradation, and preserving the physicochemical quality of food products during storage [15,16]. Incorporation of functional nanomaterials into biopolymeric matrices has emerged as an effective strategy to impart multifunctional characteristics, including antimicrobial, antioxidant, UV-blocking, and barrier properties. In this context, carbon dots (CDs) have gained remarkable attention because of their tunable surface chemistry, strong photostability, water dispersibility, low cytotoxicity, facile synthesis, and versatile biological functionalities [17,18]. Compared with conventional metal-based nanomaterials, CDs offer superior biocompatibility and lower environmental risks, making them highly attractive for food-contact applications [19,20,21,22,23,24]. Importantly, the physicochemical properties of CDs can be tailored by careful selection of carbon precursors and heteroatom dopants, enabling the development of multifunctional nanostructures with enhanced reactive oxygen scavenging ability and antimicrobial performance [25,26,27,28].

Heteroatom-doped CDs, particularly sulfur- and nitrogen-containing CDs, have demonstrated improved electron-transfer capability, abundant surface-active functional groups, and enhanced interaction with microbial cell membranes [29]. Lipoic acid is a sulfur-containing biomolecule with strong antioxidant potential and rich functional chemistry, while glycine serves as an effective nitrogen-rich precursor capable of introducing amine functionalities into the carbon framework [28]. The synergistic combination of glycine and lipoic acid offers a unique platform for generating sulfur and nitrogen-enriched CDs with improved biological and interfacial properties [30]. Although several studies have reported CD-incorporated polymeric films for food packaging applications, the utilization of glycine/lipoic acid-derived CDs within chitosan/PVA matrices remains largely unexplored. Furthermore, the influence of such multifunctional CDs on the structural, optical, barrier, antioxidant, antimicrobial, and produce preservation performance of biodegradable composite films has not been comprehensively investigated.

Herein, we report the fabrication of GLCD-reinforced chitosan/PVA composite films for active food-packaging applications. Sulfur- and nitrogen-containing carbon dots were synthesized from glycine and lipoic acid through a hydrothermal approach and subsequently incorporated into chitosan/PVA matrices via solution casting. The synthesized GLCDs and resulting composite films were characterized using TEM, FTIR, UV-visible spectroscopy, photoluminescence spectroscopy, and XPS analysis. Furthermore, the effects of GLCDs incorporation on the optical, mechanical, thermal, barrier, antioxidant, antibacterial, biodegradation, and produce-preservation properties of the films were systematically investigated. We proposed that incorporation of GLCDs would modify the physicochemical and functional properties of the chitosan/PVA matrix and contribute to the development of multifunctional biodegradable packaging materials.

## 2. Materials and Methods

### 2.1. Materials

Glycine (≥99%), lipoic acid (≥98%), poly(vinyl alcohol), and acetic acid were purchased from Sigma-Aldrich, St. Louis, MO, USA. Chitosan (degree of deacetylation 75–85%) was procured from Merck, Darmstadt, Germany. Ethanol and other analytical-grade solvents and reagents were supplied by Fisher Scientific, Hampton, NH, USA. Deionized (DI) water was utilized in all studies for the preparation of nanoparticles, polymeric solutions, and composite films.

### 2.2. Synthesis of Carbon Dots (GLCDs)

Glycine (0.50 g) and lipoic acid (0.25 g) were dissolved in 20 mL of deionized water and stirred for 30 min to obtain a homogeneous solution. The solution was transferred into a 50 mL Teflon-lined stainless-steel autoclave and heated at 180 °C for 8 h. After natural cooling to room temperature, the reaction mixture was centrifuged at 10,000 rpm for 15 min to remove large aggregates and unreacted residues. The collected supernatant was purified using a dialysis membrane with a molecular-weight cut-off (MWCO) of 3.5 kDa against deionized water for 48 h. The external dialysis medium was replaced every 10 h. The purified GLCD dispersion was collected and stored at 4 °C for further use. For solid-state characterization, the purified GLCD dispersion was freeze-dried to obtain GLCD powder. The overall yield of purified GLCDs was approximately 32%.

### 2.3. Fabrication of GLCD-Loaded Polymer Films

Chitosan/PVA composite films containing GLCDs were fabricated through a solution-casting method. Initially, 1.5 g of poly(vinyl alcohol) (PVA) was dissolved in 30 mL of deionized water at 90 °C under continuous magnetic stirring for 2 h until a clear homogeneous solution was obtained. Separately, 1.0 g of chitosan was dissolved in 30 mL of a 1% (*v*/*v*) acetic acid solution while stirring at room temperature. The prepared PVA and chitosan solutions were subsequently combined in a 1:1 volume ratio and stirred for an additional 2 h to achieve a homogeneous polymer blend. For the fabrication of GLCD-loaded composite films, different amounts of GLCDs (0, 1, 3, and 5 wt% with respect to the total polymer weight) were dispersed in 5 mL of deionized water through ultrasonication for 20 min prior to incorporation into the polymer matrix. The GLCD dispersion was slowly added into the chitosan/PVA blend under vigorous stirring followed by sonication for 15 min to ensure effective incorporation of the nanofillers within the polymer network. In this work, CP0, CP1, CP2, and CP3 correspond to chitosan/PVA films containing 0, 1, 3, and 5 wt% GLCDs, respectively. For each formulation, approximately 25 mL of the film-forming solution was cast into a polystyrene Petri dish with a diameter of 90 mm. The films were dried at 70 °C in a hot-air oven for 10 h to ensure complete solvent evaporation. After drying, the films were carefully peeled off and conditioned at 25 °C and 50% relative humidity for at least 24 h prior to characterization and application studies. Film thickness was measured using a digital micrometer at five different locations, and the average thickness was found to be approximately 85 ± 3 μm. Film thickness was measured using a digital micrometer at five random positions on each film, and the average value was reported as mean ± standard deviation.

### 2.4. Biodegradation Assay

Biodegradation behavior of the films was evaluated using a soil-burial degradation test. Film specimens with dimensions of 20 mm × 20 mm were accurately weighed (W_0_) and buried in natural soil at a depth of approximately 5 cm. The soil moisture content was maintained at approximately 60–70% by periodic addition of deionized water, and the experiment was conducted at 25 ± 2 °C. At predetermined intervals, the samples were carefully removed from the soil, gently washed with deionized water to remove adhering soil particles, and dried in a hot-air oven at 50 °C until a constant weight was achieved. The dried samples were then weighed (W_t_), and the percentage weight loss was calculated. All experiments were performed in triplicate (*n* = 3), and the results are presented as mean ± standard deviation.

### 2.5. Water Vapor Permeability Measurement

Water vapor permeability (WVP) of the films was determined using a gravimetric cup method based on ASTM E96. Briefly, film samples were sealed over permeation cups containing anhydrous calcium chloride (0% RH), while the cups were placed in a controlled environment maintained at 25 ± 2 °C and 75 ± 2% relative humidity. The effective exposed area for vapor transmission was approximately 7.07 cm^2^. The average film thickness was 85 ± 3 μm and was measured using a digital micrometer at five different locations.

The weight gain of each cup was recorded at predetermined intervals, and the water vapor transmission rate (WVTR) was calculated from the slope of weight gain versus time. The WVP was calculated using the following equation:

WVP = (WVTR × L)/ΔP(1)

where WVTR is the water vapor transmission rate (g m^−2^ h^−1^), L is the film thickness (m), and ΔP is the water vapor partial-pressure difference across the film (Pa). All measurements were performed in triplicate (*n* = 3), and the results are presented as mean ± standard deviation.

### 2.6. Antioxidant Activity Measurement

Detailed experimental procedures for the DPPH and ABTS antioxidant assays are included in the Supplementary Materials. These protocols were used for all antioxidant activity measurements reported in this study.

### 2.7. Cytocompatibility and Cell Proliferation Assay

Cytocompatibility and cell proliferation experiments were conducted using the established NIH/3T3 mouse embryonic fibroblast cell line (ATCC CRL-1658; American Type Culture Collection, Manassas, VA, USA). Comprehensive experimental details related to the MTT assay and live/dead cell staining are available in the Supplementary Materials. These methods were employed to evaluate the cytocompatibility and cell proliferation behavior of the fabricated films.

### 2.8. Produce Preservation and Antibacterial Evaluation

Detailed experimental procedures for the produce preservation study and antibacterial assays are described in the Supplementary Materials. All experiments were performed using at least three independent measurements unless otherwise stated, and the results are presented as mean ± standard deviation. Detailed experimental procedures, instrument settings, temperature ranges, sample preparation protocols, and additional characterization methodologies are provided in the Supplementary Materials.

The antibacterial activity of the films was evaluated against *Escherichia coli* (ATCC 25922) using the agar disc diffusion method. A bacterial suspension with an approximate concentration of 1 × 10^6^ CFU mL^−1^ (0.5 McFarland standard) was uniformly spread onto nutrient agar plates.

Circular film discs (6 mm in diameter) were placed on the inoculated agar surface and incubated at 37 °C for 24 h. A PVA-only film was used as the negative control, while CP0, consisting of the chitosan/PVA film without GLCDs, served as the base-film control. A standard antibiotic disc (streptomycin) was used as the positive control. Following incubation, the inhibition-zone diameters were measured in millimeters. All experiments were performed in triplicate (*n* = 3), and the results are reported as mean ± standard deviation.

### 2.9. Release Study

Release studies were performed to evaluate the release behavior of GLCDs-associated species from the chitosan/PVA nanocomposite films in different food-simulant media. Film specimens (20 mm × 20 mm) were accurately weighed and immersed separately in 20 mL of distilled water, 10% (*v*/*v*) ethanol, 50% (*v*/*v*) ethanol, and 3% (*v*/*v*) acetic acid at room temperature (25 ± 2 °C). At predetermined time intervals, aliquots were withdrawn and analyzed using a UV-visible spectrophotometer at the characteristic absorption wavelength of GLCDs. At predetermined time intervals, aliquots were withdrawn and analyzed using a UV-visible spectrophotometer at 278 nm, corresponding to the characteristic absorption band of GLCDs. A calibration curve was prepared using purified GLCD dispersions of known concentrations, and the concentration of released GLCD-associated species was calculated from the corresponding calibration equation. An equal volume of fresh medium was replenished after each sampling interval to maintain a constant solution volume. An equal volume of fresh medium was replenished after each sampling to maintain a constant solution volume.

The cumulative release percentage was calculated according to:

Release (%) = (M_t_/M_∞_) × 100 (2)

where M_t_ is the cumulative amount of released GLCD-associated species at time t and M_∞_ is the maximum cumulative release amount determined from the equilibrium (plateau) region of the release profile. All release experiments were performed in triplicate (*n* = 3), and the results are presented as mean ± standard deviation.

The selected media were used to simulate different food environments, including aqueous (water), alcoholic (10% and 50% ethanol), and acidic (3% acetic acid) food systems. The release profiles were monitored for up to 120 min to evaluate the influence of solvent composition on the release behavior of the active components from the film matrix.

### 2.10. Statistical Analysis

All experiments were performed in triplicate unless otherwise stated, and the results are presented as mean ± standard deviation (SD). Statistical analyses were conducted using one-way analysis of variance (ANOVA) to evaluate differences among experimental groups. When significant differences were observed, Tukey’s multiple comparison post-hoc test was performed to identify pairwise differences between groups. Statistical significance was considered at *p* < 0.05. For mechanical testing, at least five specimens (*n* = 5) were analyzed for each film formulation. The reported values represent the mean of independent measurements, and error bars in the figures correspond to standard deviation.

## 3. Results and Discussion

### 3.1. Synthesis and Characterization of GLCDs

**Figure 1:**
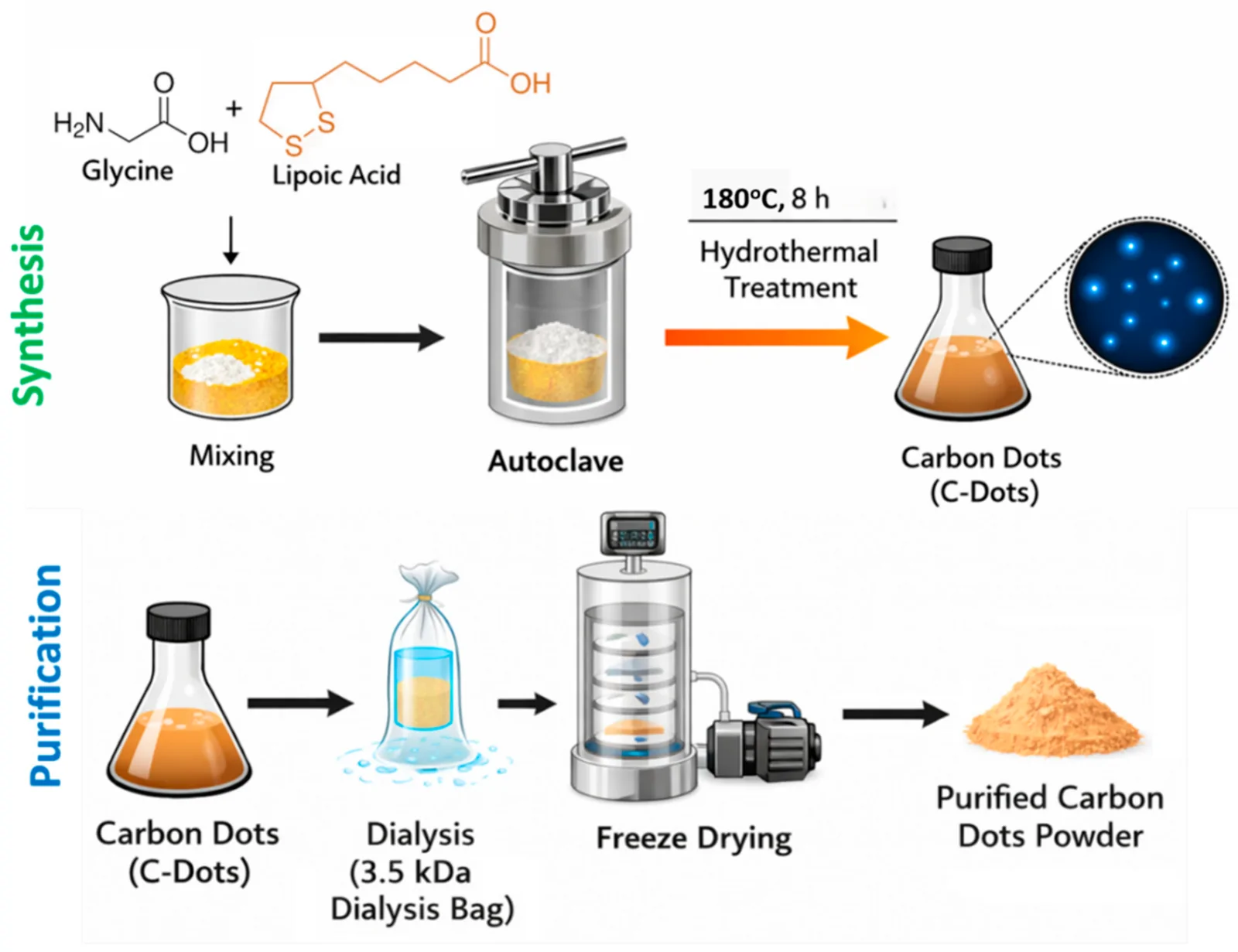
Schematic representation of the synthesis and purification process of GLCDs via hydrothermal treatment, followed by purification to obtain purified GLCDs powder.

Figure 1 schematically illustrates the synthesis and purification process of glycine-lipoic acid-derived carbon dots (GLCDs) prepared via a one-pot hydrothermal approach. Glycine and lipoic acid served as carbon, nitrogen, and sulfur precursors for the formation of heteroatom-doped CDs. During hydrothermal treatment, dehydration, condensation, and carbonization reactions led to the formation of nanoscale carbonaceous structures enriched with surface functional groups. Post-synthesis purification was carried out through centrifugation and dialysis to remove unreacted precursors, small molecular impurities, and larger carbonaceous aggregates. The purified GLCD dispersion was subsequently freeze-dried to obtain dry GLCD powder for further characterization and composite film fabrication. The purified GLCDs were further incorporated into chitosan/PVA matrices through solution casting to fabricate nanocomposite films. Owing to the abundant hydroxyl, amino, and carboxyl surface functionalities of GLCDs, strong intermolecular hydrogen bonding interactions with chitosan and PVA chains are expected, promoting effective incorporation of nanofiller dispersion within the polymer matrix. Such interactions may enhance the structural integrity and multifunctional properties of the composite films. The prepared films exhibited an average thickness of approximately 85 ± 3 μm, and no significant differences were observed among CP0, CP1, CP2, and CP3, indicating that GLCDs incorporation had minimal influence on film thickness. This thickness range is comparable to that reported for many commercial flexible and biodegradable food-packaging films (20–150 μm), suggesting that the developed films are suitable for practical packaging applications.

**Figure 2:**
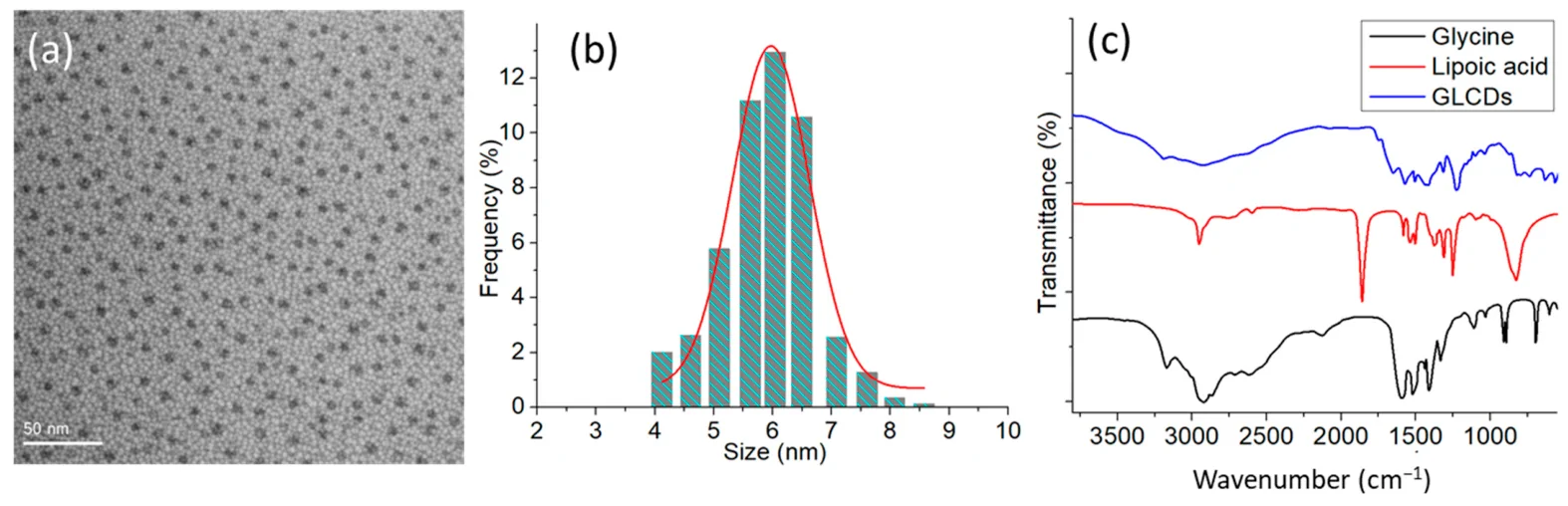
(**a**) Particle size distribution histogram of GLCDs showing an average particle diameter of 5.8 ± 1.2 nm (*n* = 50 particles). (**b**) Particle size distribution of GLCDs. (**c**) FTIR spectra of GLCDs, glycine, lipoic acid.

The TEM image (Figure 2a) of the synthesized GLCDs, revealed the formation of well distributed quasi-spherical nanoparticles. The particle size distribution histogram indicates that the GLCDs have an average particle diameter of around 5.8 ± 1.2 nm, with the majority of particles ranging from 4.5 to 7.0 nm. The FTIR spectra of glycine, lipoic acid, and the synthesized GLCDs are shown in Figure 2c to examine the surface functional groups and chemical composition of the prepared CDs. Glycine displayed a broad absorption band in the 3200–3400 cm^−1^ region, which is associated with the stretching vibrations of O–H and N–H groups. The absorption peaks observed at approximately 2920 and 2850 cm^−1^ correspond to the stretching vibrations of aliphatic C–H bonds. In addition, characteristic bands located near 1600 and 1400 cm^−1^ were assigned to the asymmetric and symmetric stretching modes of carboxylate (–COO^−^) groups, respectively [31]. Lipoic acid showed characteristic absorption peaks near 2925 and 2855 cm^−1^ associated with aliphatic C–H stretching vibrations, whereas the strong band around 1690 cm^−1^ corresponded to C=O stretching of carboxylic acid groups. Additional peaks observed within 900–1400 cm^−1^ were attributed to C–S, C–O, and C–C stretching vibrations [32]. Post-hydrothermal treatment, GLCDs exhibited a broad absorption band centered between 3300 and 3400 cm^−1^, suggesting the presence of hydroxyl and amino groups on the surface of the CDs [28,33]. The appearance of bands near 1630 cm^−1^ was assigned to C=O stretching and/or C=C skeletal vibrations of partially graphitized carbon domains. Peaks observed around 1380–1450 cm^−1^ were associated with C–N and C–O stretching vibrations, confirming successful incorporation of nitrogen-containing functionalities from glycine [34]. Furthermore, absorption features within 1050–1250 cm^−1^ suggested the presence of C–O–C and C–S related surface groups, indicating sulfur incorporation from lipoic acid into the carbon framework [35,36]. The presence of these oxygen, nitrogen, and sulphur containing functional groups are advantageous for improving aqueous dispersibility and fluorescence behavior of the GLCDs. Overall, the TEM and FTIR analyses collectively confirm the successful synthesis of uniformly dispersed, heteroatom doped GLCDs with nanoscale dimensions and abundant surface active functional groups suitable for incorporation into chitosan/PVA composite films. Although the observed FTIR peak shifts are consistent with interactions between GLCDs and the polymer matrix, direct confirmation of nanoparticle dispersion, aggregation state, or specific intermolecular interaction mechanisms would require complementary morphological analyses such as SEM, TEM, or elemental mapping.

**Figure 3:**
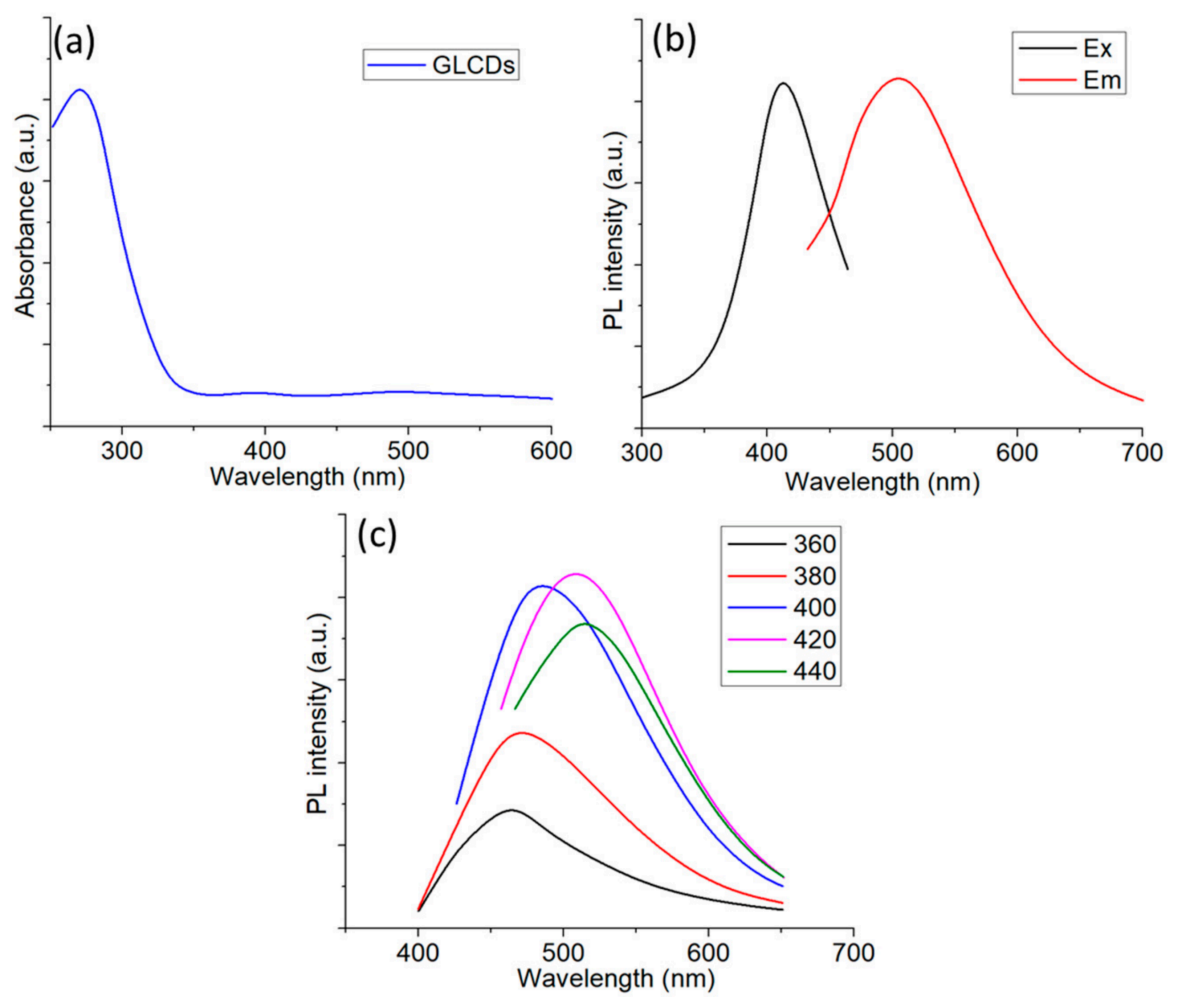
(**a**) UV-vis absorption spectrum, (**b**) photoluminescence excitation and emission spectra, and (**c**) excitation-dependent emission spectra of the GLCDs.

The optical properties of the GLCDs were investigated using UV-visible absorption and photoluminescence (PL) spectroscopy (Figure 3a). As illustrated in Figure 3a, the UV–Vis absorption spectrum of the GLCDs displays a prominent absorption peak in the 270–280 nm region. This absorption is assigned to the π → π* electronic transition of aromatic sp^2^-hybridized C=C/C–C domains generated during the hydrothermal carbonization process [37]. A weak shoulder extending into the visible region (~350–500 nm) is also observed, which is associated with n → π* transitions of surface functional groups such as C=O, C=N, and sulfur-containing moieties originating from glycine and lipoic acid [38,39]. The PL excitation and emission spectra of GLCDs are presented in Figure 3b. The maximum excitation wavelength is located at approximately 420 nm, while the corresponding emission peak appears at around 510 nm, resulting in a large Stokes shift of nearly 90 nm. Figure 3c shows the excitation-dependent emission behavior of the GLCDs recorded at excitation wavelengths ranging from 360 to 440 nm. With increasing excitation wavelength, the emission intensity initially increases and reaches a maximum at 420 nm, followed by a slight decrease at higher excitation wavelengths. Meanwhile, the emission maximum exhibits a slight red shift from approximately 490 nm to 525 nm. This excitation-dependent PL behavior is a characteristic feature of carbon dots and is generally attributed to the presence of multiple emissive sites, surface defect states, and a distribution of particle sizes. Different excitation wavelengths selectively activate different surface energy levels, resulting in tunable emission characteristics [40]. These optical properties are advantageous for incorporating GLCDs into chitosan/PVA matrices to impart fluorescence functionality to the resulting films.

**Figure 4:**
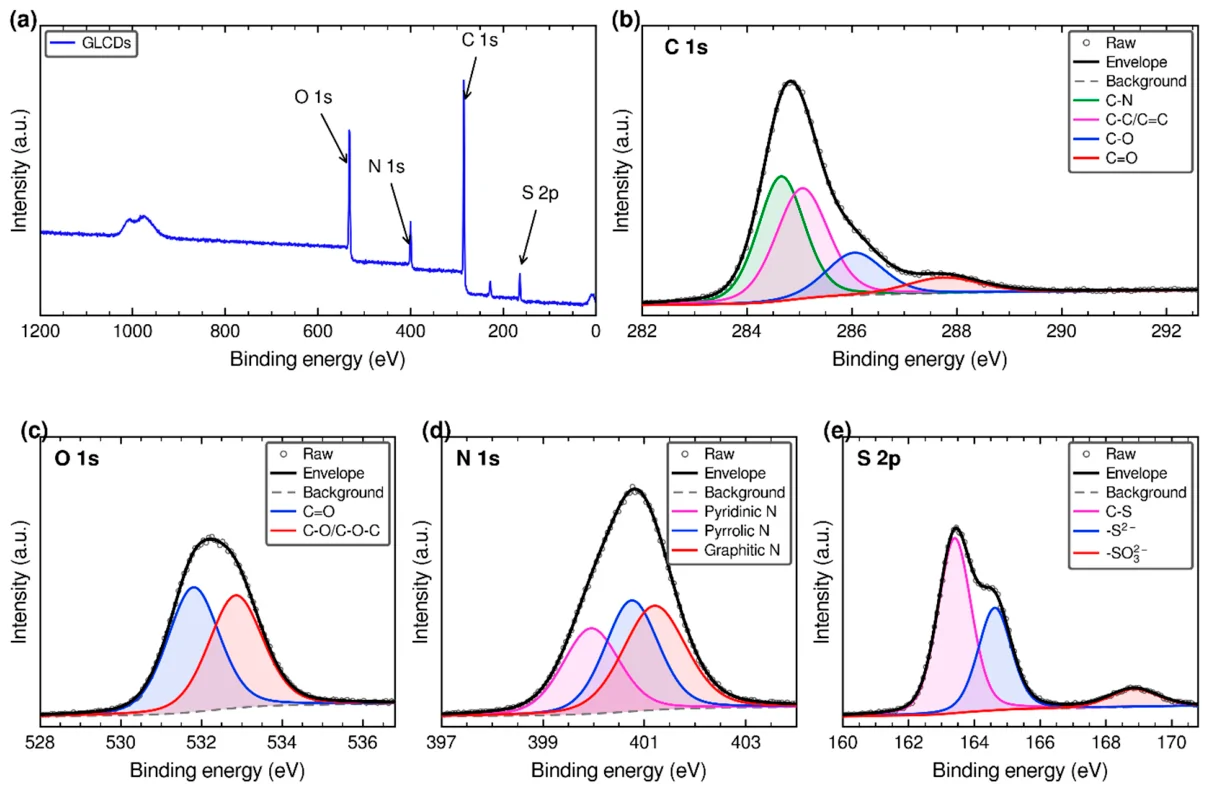
XPS characterization of GLCDs showing (**a**) survey spectrum and high-resolution spectra of (**b**) C1s, (**c**) O1s, (**d**) N1s, and (**e**) S2p. Experimental spectra are presented together with the fitted components used for peak deconvolution. The results confirm the presence of carbon-, oxygen-, nitrogen-, and sulfur-containing surface functionalities on the GLCDs.

The elemental composition and chemical bonding environment of the GLCDs were characterised using X-ray photoelectron spectroscopy (XPS). As shown in the survey spectrum (Figure 4a), distinct signals corresponding to carbon, oxygen, nitrogen, and sulfur are observed. Quantitative analysis of the XPS survey spectrum revealed that the GLCDs consisted of C (72.4 at.%), O (18.1 at.%), N (6.7 at.%), and S (2.8 at.%). The characteristic peaks located at approximately 285.0 eV, 532.0 eV, 400.5 eV, and 164.0 eV are assigned to the C1s, O1s, N1s, and S2p, respectively, confirming the presence of carbon, oxygen, nitrogen, and sulfur functionalities within the GLCDs structure. The coexistence of nitrogen and sulfur indicates the presence of nitrogen- and sulfur-containing surface functionalities associated with the synthesized GLCDs. The high-resolution C1s spectrum (Figure 4b) was deconvoluted into four distinct components located at approximately 284.5, 285.1, 286.2, and 287.8 eV. These peaks are assigned to C–C/C=C, C–N/C–S, C–O, and C=O bonding environments, respectively, indicating the presence of diverse carbon-containing functional groups on the surface of the GLCDs [35,41]. The O1s spectrum (Figure 4c) exhibits two major peaks located at approximately 531.6 eV and 533.0 eV, which are assigned to C=O and C–O/C–O–C groups, respectively [42]. As presented in Figure 4d, the high-resolution N1s spectrum can be fitted into three distinct components centered at approximately 399.8, 400.6, and 401.2 eV. These peaks are attributed to pyridinic N, pyrrolic N, and C=N–C nitrogen species, respectively, confirming the incorporation of different nitrogen functionalities within the GLCD framework [43]. The S2p spectrum (Figure 4e) further confirms sulfur incorporation within the GLCDs. The peaks observed at approximately 163.2–164.5 eV are attributed to C–S and S^2−^ species, while a weak peak at around 168.5–169.0 eV corresponds to oxidized sulfur species (–SO_3_^−^) [44]. These sulfur-containing functionalities originate from lipoic acid and provide additional surface-active sites that can influence charge transfer and photoluminescence behavior. The XPS analysis confirms the presence of carbon-, oxygen-, nitrogen-, and sulfur-containing chemical species on the GLCD surface. These findings are consistent with the formation of nitrogen- and sulfur-functionalized carbon dots and support the successful introduction of heteroatom-containing surface functionalities during synthesis. The abundant surface functionalities and heteroatom dopants generate diverse surface states and defect sites, which are responsible for the favourable optical properties and excellent compatibility of the GLCDs with the chitosan/PVA matrix.

### 3.2. Fabrication of GLCDs Based Nanocomposite Films

**Figure 5:**
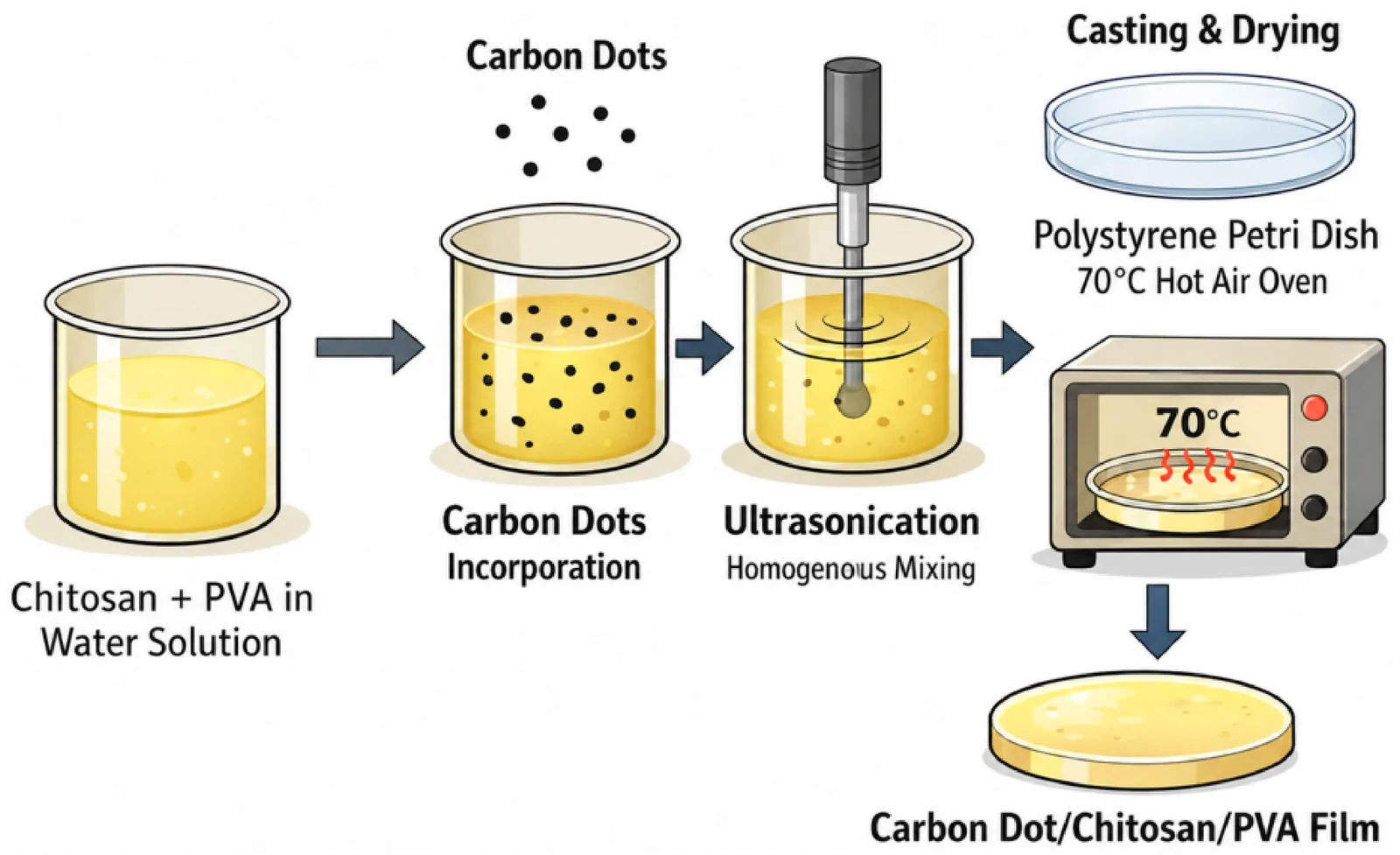
Schematic illustration of the fabrication of GLCD-incorporated chitosan/PVA nanocomposite films through solution casting. The process involves preparation of the chitosan/PVA polymer blend, addition of glycine-lipoic acid derived carbon dots (GLCDs), ultrasonication-assisted mixing and incorporation, followed by casting and thermal drying to obtain multifunctional films for active food-packaging applications.

GLCD-incorporated chitosan/PVA nanocomposite films were successfully fabricated through a facile solution-casting approach, as schematically illustrated in Figure 5. The incorporation of GLCDs into the chitosan/PVA matrix resulted in flexible and optically transparent films without visible macroscopic phase separation, suggesting good compatibility between the nanofillers and the polymer network. The abundant oxygen-, nitrogen-, and sulfur-containing surface functional groups present on the GLCDs facilitated strong intermolecular interactions, particularly hydrogen bonding, with the hydroxyl and amino groups of the chitosan/PVA matrix. Such interfacial interactions played a critical role in contributing to the overall integrity of the polymer matrix and stability of the composite films. The ultrasonication-assisted mixing process was intended to facilitate incorporation of GLCDs into the polymer matrix, facilitating GLCDs distribution during film preparation and enabling effective stress transfer within the composite structure. The resulting films appeared visually uniform without visible macroscopic phase separation and enhanced structural integrity, suggesting successful formation of a well-integrated nanocomposite network. In addition, the incorporation of GLCDs imparted a faint yellowish appearance to the films while maintaining sufficient optical transparency, which is highly desirable for food packaging applications where visual inspection of packaged products is important. The presence of GLCDs within the polymeric framework is expected to significantly influence the physicochemical and functional properties of the films [45]. Owing to their nanoscale dimensions and multifunctional surface chemistry, GLCDs can enhance intermolecular interactions within the chitosan/PVA matrix, thereby improving mechanical strength, thermal stability, UV-shielding performance, and barrier characteristics. Furthermore, sulfur- and nitrogen-rich GLCDs provide active antioxidant and antimicrobial functionalities through radical scavenging and reactive oxygen species-mediated antibacterial mechanisms [46]. The combination of these multifunctional properties highlights the potential of the developed GLCDs-based nanocomposite films as sustainable active food packaging materials capable of prolonging the shelf life and maintaining the freshness and quality of perishable produce.

### 3.3. Structural and Optical Characterization of GLCD-Based Nanocomposite Films

**Figure 6:**
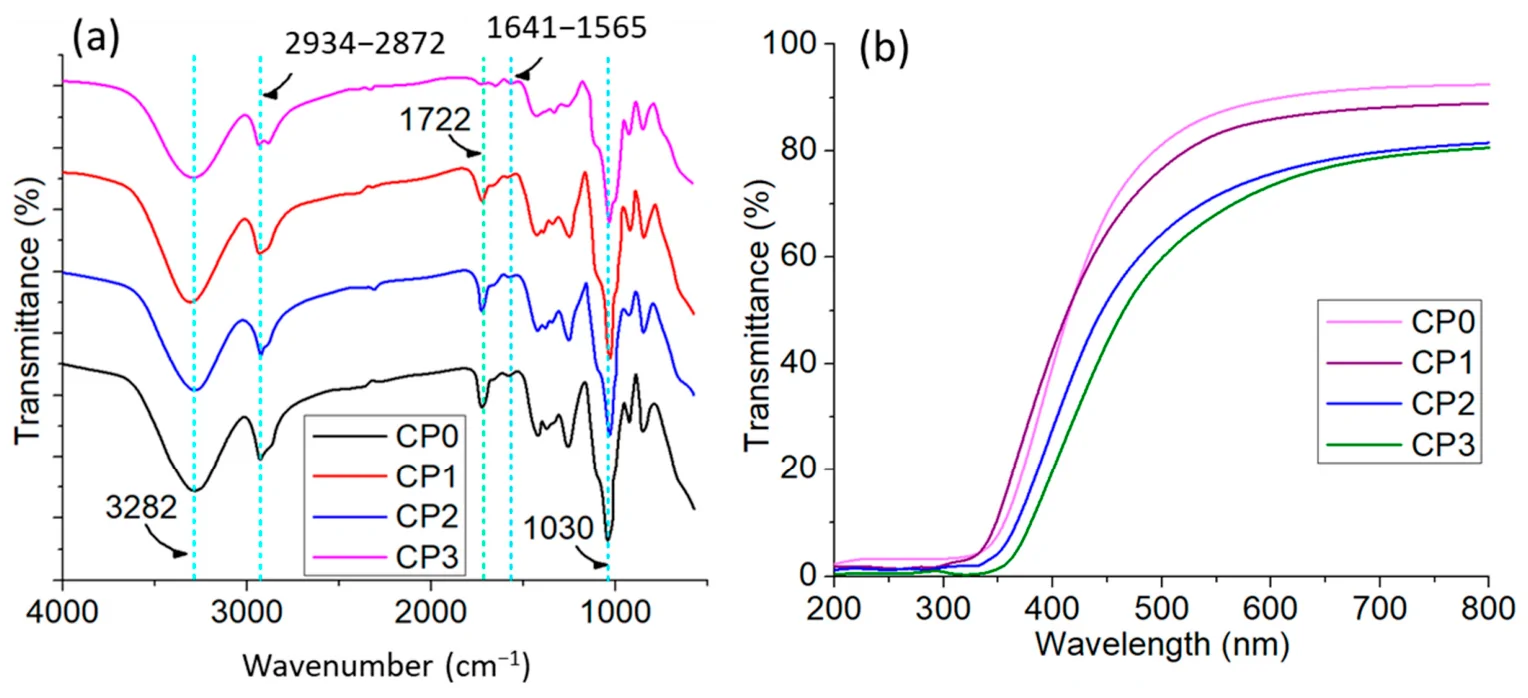
(**a**) FTIR spectra of pristine and GLCD-incorporated chitosan/PVA nanocomposite films containing 0 wt% (CP0), 1 wt% (CP1), 3 wt% (CP2), and 5 wt% (CP3) GLCDs showing intermolecular interactions and functional group variations after GLCD incorporation. (**b**) UV–visible transmittance spectra of the films demonstrating the influence of GLCD loading on optical transparency and UV-blocking performance.

The chemical interactions between the GLCDs and the chitosan/PVA polymer matrix were investigated using FTIR spectroscopy, and the corresponding spectra are presented in Figure 6a. The FTIR spectrum of the pristine chitosan/PVA (CP0) film displays a broad absorption band centered at approximately 3282 cm^−1^, which arises from the overlapping stretching vibrations of hydroxyl (–OH) and amino (–NH) functional groups originating from both the chitosan and PVA matrices [12]. Upon incorporation of GLCDs, this band gradually shifted toward lower wavenumbers with a noticeable reduction in intensity, consistent with the presence of intermolecular interactions between GLCD surface functional groups and the polymer chains. The peaks in the range of 2934–2872 cm^−1^ relate to the asymmetric and symmetric stretching vibrations of aliphatic C–H groups in the polymer backbone [47]. A distinct absorption peak around 1722 cm^−1^ became more pronounced after GLCD incorporation, which can be assigned to the stretching vibration of carbonyl (C=O) groups originating from oxygen-containing functionalities on the GLCDs surface. This observation confirms the successful integration of GLCDs into the polymeric matrix. Additionally, the absorption bands within the region of 1641–1565 cm^−1^ are associated with amide I and amide II vibrations as well as N–H bending modes of chitosan. Slight peak shifting and broadening in this region may indicate interactions between GLCD surface functionalities and the polymer chains. The intense peak observed near 1030 cm^−1^ corresponds to C–O–C and C–O stretching vibrations of the polysaccharide backbone, which also exhibited minor spectral changes after GLCD addition, indicating modification of the local polymeric environment due to nanofiller incorporation.

The transparency of the fabricated films was assessed by UV–visible spectroscopy, and the corresponding spectra are presented in Figure 6b. The pristine CP0 film exhibited high optical transparency with approximately 90–92% transmittance in the visible region. After incorporation of GLCDs, a gradual decrease in transmittance was observed with increasing nanofiller concentration. Specifically, the transmittance decreased to approximately 88%, 81%, and 80% for CP1, CP2, and CP3 films, respectively, at 800 nm. This reduction in transparency can be attributed to enhanced light absorption and scattering induced by the GLCDs incorporated within the polymer matrix. Notably, all GLCD-containing films exhibited significantly reduced transmittance in the UV region (200–400 nm), indicating improved UV-blocking capability. Such behavior is highly beneficial for active food packaging applications because UV irradiation can accelerate lipid oxidation, nutrient degradation, and microbial spoilage in food products. The enhanced UV-shielding performance of the nanocomposite films is mainly associated with the π-π* and n-π* electronic transitions of conjugated carbon domains and surface functional groups present in the GLCDs. Despite the decrease in optical transparency at higher GLCD loading, the films maintained sufficient visible-light transmittance for practical packaging applications, allowing visual inspection of packaged produce while simultaneously providing protection against harmful UV radiation.

### 3.4. Mechanical, Thermal, Biodegradation, and Barrier Properties of Nanocomposite Films

**Figure 7:**
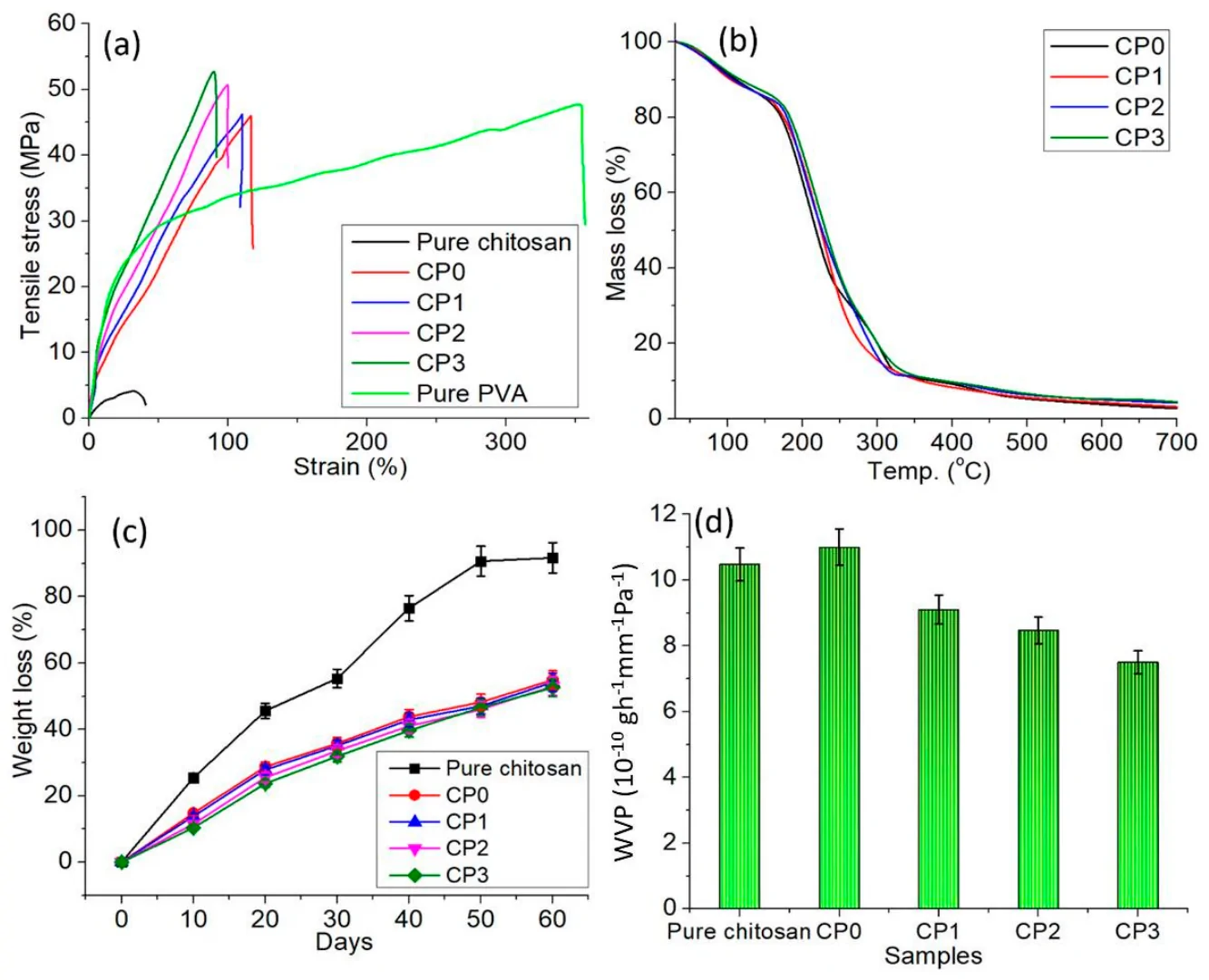
Mechanical, thermal, biodegradation, and barrier properties of films containing 0 wt% (CP0), 1 wt% (CP1), 3 wt% (CP2), and 5 wt% (CP3) GLCDs. (**a**) Tensile stress–strain curves of pristine and GLCDs-incorporated chitosan/PVA nanocomposite films. (**b**) Thermogravimetric analysis (TGA) curves showing the thermal stability of the films. (**c**) Biodegradation behavior of the films evaluated through weight loss over 60 days. (**d**) Water vapor permeability (WVP) of the fabricated films demonstrating improved moisture barrier performance after GLCD incorporation. Data are presented as mean ± SD (*n* = 3).

The mechanical properties of the fabricated films were evaluated through tensile testing, and the corresponding stress–strain curves are presented in Figure 7a. Pure chitosan exhibited very poor mechanical performance, showing a tensile strength of approximately 4 MPa and an elongation at break of approximately 39%, reflecting its inherently brittle nature. In contrast, blending with PVA significantly improved the mechanical behavior of the films. The pristine chitosan/PVA film (CP0) exhibited a tensile strength of approximately 45.5 MPa with an elongation at break of about 117%. Upon incorporation of GLCDs, the tensile properties were further modified. Tensile strength and elongation-at-break values estimated from the stress–strain curves indicate that CP3 exhibited the highest tensile strength (~52.5 MPa), whereas pure PVA showed the highest elongation at break (~355%). Among the GLCD-incorporated films, CP2 exhibited the highest Young’s modulus (~100 MPa), indicating the most effective reinforcement of the chitosan/PVA network. Tensile strength increased progressively with increasing GLCDs loading, rising from approximately 45.5 MPa for CP0 to 52.5 MPa for CP3. The enhancement in mechanical performance may be attributed to favorable interactions between the surface functional groups of GLCDs and the chitosan/PVA matrix, which facilitate stress transfer across the composite structure. Tensile strength, elongation-at-break, and Young’s modulus values are summarized in Table S1. The elongation at break decreased from approximately 117% for CP0 to 109% and 99% for CP1 and CP2, respectively, indicating a gradual reduction in film flexibility at moderate GLCD loadings. CP3 exhibited elongation at break to approximately 89%, while maintaining a relatively high tensile strength of approximately 52.5 MPa. Overall, GLCD incorporation influenced the flexibility of the films in a concentration-dependent manner. The resulting mechanical response reflected a balance between tensile strength and ductility, indicating that the incorporation of GLCDs effectively modulated the mechanical behavior of the chitosan/PVA films [48]. Improvements in mechanical properties have been reported for carbon-dot-reinforced polymer nanocomposites and are commonly attributed to favorable interactions between carbon-dot surface groups and polymer chains, which may strengthen the polymer network and facilitate stress transfer [49].

The thermal stability of the films was investigated using thermogravimetric analysis (TGA), as shown in Figure 7b. All films exhibited a minor initial weight loss below 150 °C, corresponding to the evaporation of physically adsorbed moisture. The major thermal degradation stage occurred within the temperature range of approximately 200–320 °C, which is associated with decomposition of the polymer backbone and cleavage of glycosidic and carbonaceous structures. The GLCD-incorporated films exhibited slightly improved thermal resistance compared with the pristine CP0 film, particularly at elevated temperatures. This improvement may be associated with interactions between GLCDs and the polymer matrix as well as the inherent thermal stability of the carbonaceous nanofiller, although direct mechanistic evidence was not investigated in the present study, which can restrict polymer chain motion and delay thermal decomposition.

The biodegradation behavior of the films was evaluated over a 60-day period, and the results are presented in Figure 7c. Pure chitosan films exhibited rapid degradation, reaching approximately 90% weight loss after 60 days. In contrast, the chitosan/PVA blend films (CP0–CP3) showed lower weight-loss values of approximately 52–55% over the same period. This observation suggests that incorporation of PVA substantially altered the degradation behavior of the films. However, only relatively small differences were observed among CP0, CP1, CP2, and CP3, indicating that the influence of GLCDs incorporation on overall biodegradation was limited under the present experimental conditions. Therefore, the current results do not allow definitive attribution of the reduced degradation behavior to GLCDs alone. Additional studies involving detailed structural characterization and statistical analysis would be required to establish the specific role of GLCDs in controlling degradation kinetics [50]. Such controlled biodegradation behavior is advantageous for active food packaging applications because it provides sufficient structural stability during storage while maintaining environmental degradability after disposal. The biodegradation results represent the average of three independent measurements and are reported as mean ± standard deviation.

The water vapor permeability (WVP) of the films is shown in Figure 7d. The pristine chitosan/PVA film (CP0) exhibited a WVP value of approximately 11.0, whereas the GLCD-incorporated films demonstrated progressively lower permeability with increasing GLCDs content. The WVP values decreased to approximately 9.0, 8.4, and 7.5 for CP1, CP2, and CP3, respectively, with CP3 exhibiting the lowest permeability. The CP3 film exhibited the lowest WVP value of approximately 7.5 × 10^−10^ gh^−1^ mm^−1^ Pa^−1^. The improved barrier performance may be associated with the presence of GLCDs within the polymer matrix, which could increase the diffusion path length for water transport and promote formation of a denser polymer network. Additionally, the strong hydrogen-bonding interactions between GLCDs and polymer chains contributed to the formation of a denser network structure, thereby restricting moisture transport through the films. Enhanced water vapor barrier properties are highly beneficial for food packaging applications because they help maintain food freshness and reduce moisture-induced spoilage.

### 3.5. Surface Wettability and Antioxidant Activity of the Nanocomposite Films

**Figure 8:**
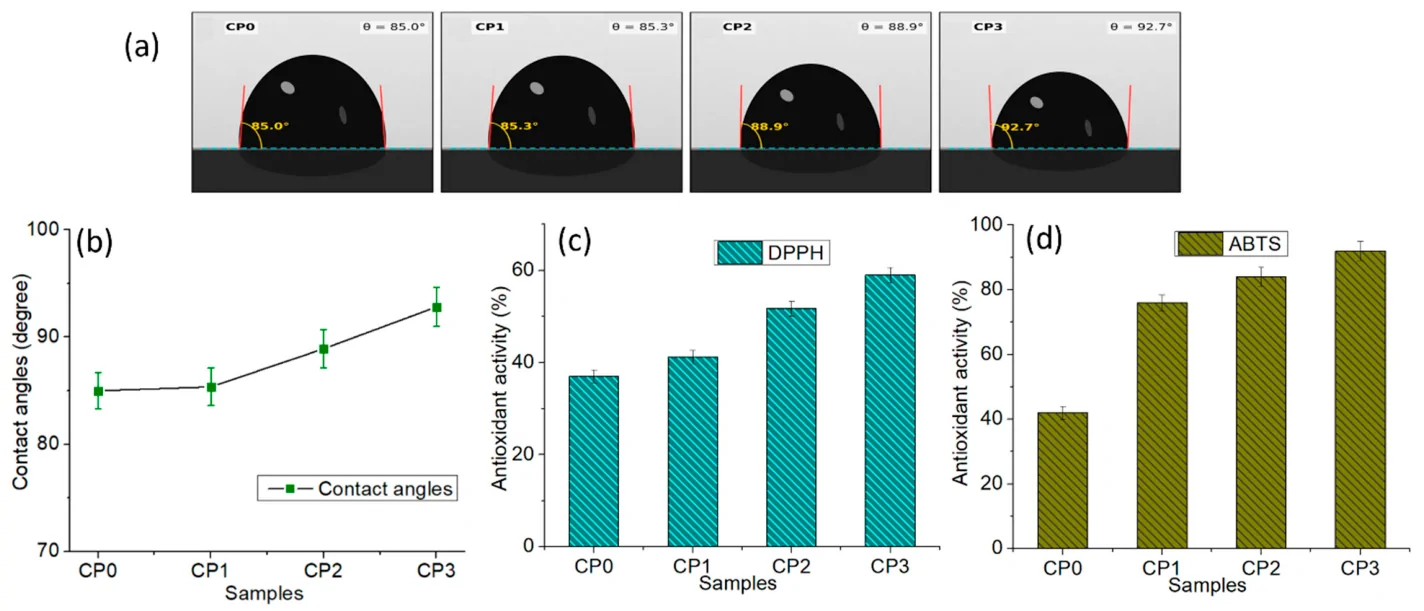
(**a**) Representative water-droplet images of 0 wt% (CP0), 1 wt% (CP1), 3 wt% (CP2), and 5 wt% (CP3) GLCDs loaed films used for contact-angle determination. (**b**) Water contact angle of the fabricated films. Data are presented as mean ± SD (*n* = 3). (**c**) DPPH and (**d**) ABTS assay of the fabricated films demonstrating improved antioxidant performance with increasing GLCD concentration. Data are presented as mean ± SD (*n* = 3). Statistical significance was determined at *p* < 0.05.

The wettability of the films was assessed via water contact angle measurements (Figure 8). Representative droplet images are shown in Figure 8a. The gradual increase in contact angle from approximately 85° for CP0 to 93° for CP3 indicates an increase in surface hydrophobicity following GLCD incorporation (Figure 8b). The pristine chitosan/PVA film (CP0) exhibited a contact angle of approximately 85°, indicating the moderately hydrophilic nature of the polymer matrix. Upon incorporation of GLCDs, the contact angle gradually increased, reaching nearly 93° for the CP3 film. This increase in contact angle suggests enhanced surface hydrophobicity of the composite films after GLCD incorporation. The improved hydrophobic behavior can be attributed to the formation of a more compact polymeric network and the strong intermolecular interactions between GLCDs and the chitosan/PVA matrix, which reduced the availability of free hydrophilic functional groups on the film surface. Enhanced hydrophobicity is advantageous for food packaging applications because it can improve moisture resistance and reduce water-induced deterioration of packaged products.

The antioxidant performance of the films was investigated using DPPH and ABTS radical scavenging assays, as shown in Figure 8c,d. The pristine CP0 film demonstrated relatively low antioxidant activity with approximately 37% DPPH radical scavenging efficiency. After incorporation of GLCDs, the antioxidant activity significantly increased in a concentration-dependent manner, reaching nearly 60% for the CP3 film. Similarly, the ABTS radical scavenging activity increased from approximately 42% for CP0 to nearly 90% for CP3. The remarkable enhancement in antioxidant activity can be attributed to the abundant surface-active functional groups and electron-rich conjugated carbon domains present in the GLCDs. The sulfur- and nitrogen-containing functionalities introduced from glycine and lipoic acid precursors likely contributed to efficient free radical scavenging through electron transfer and hydrogen donation mechanisms. Moreover, the nanoscale incorporation of GLCDs within the polymer matrix enabled greater exposure of active surface sites, facilitating improved interaction with reactive radical species. The superior antioxidant performance of the GLCD-incorporated films is highly beneficial for active food packaging applications because oxidative degradation is one of the primary causes of quality deterioration in fresh produce and food products during storage.

### 3.6. Controlled Release, Produce Preservation, and Antibacterial Performance of the Nanocomposite Films

**Figure 9:**
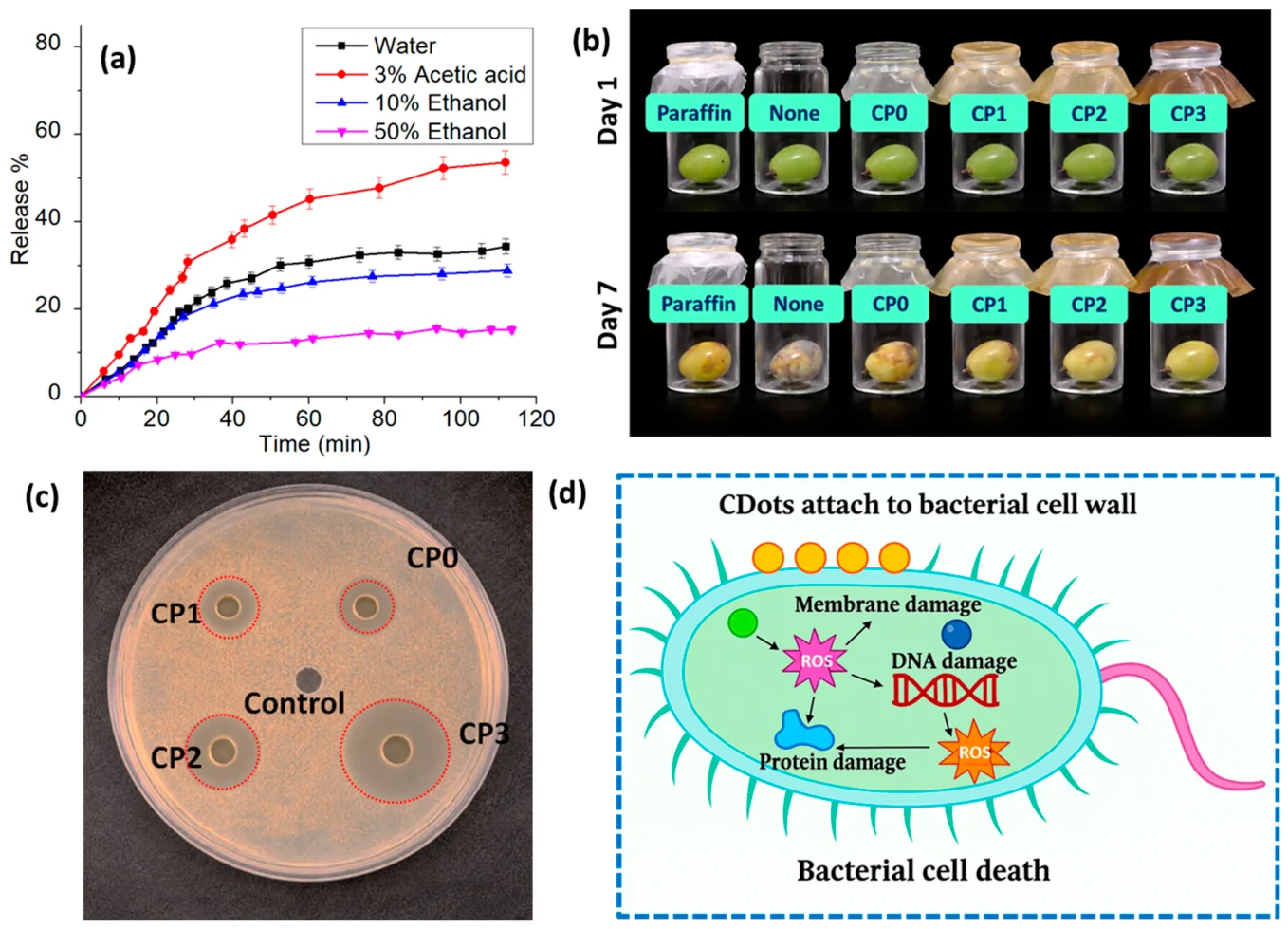
(**a**) Release profiles of active components from GLCDs-incorporated nanocomposite films containing 5 wt% (CP3) GLCDs in different solvent environments. Data are presented as mean ± SD (*n* = 3). Statistical significance was determined at *p* < 0.05. (**b**) Photographic images showing the visual appearance of unwrapped and film-wrapped produce samples after 7 days of storage. (**c**) Antibacterial activity of CP0, CP1, CP2, and CP3 films against *E. coli* determined by the agar diffusion method. Inhibition-zone diameters are reported as mean ± SD (*n* = 3). (**d**) Hypothesized antibacterial mechanism of GLCDs based on literature reports, involving membrane interaction and potential ROS-mediated antibacterial effects.

The release behavior of GLCD-associated species from the GLCD-incorporated nanocomposite film (CP3) was investigated in different solvent environments (Figure 9a). The release profile reflects the diffusion of GLCD-related soluble components from the chitosan/PVA matrix into the surrounding medium and was monitored by UV-visible spectroscopic analysis. The films exhibited solvent-dependent release characteristics with the highest release observed in 3% acetic acid solution, followed by water and 10% ethanol, whereas the lowest release was obtained in 50% ethanol. Specifically, the cumulative release reached approximately 52% in 3% acetic acid after 120 min, while only nearly 15% release was observed in 50% ethanol under the same conditions. The enhanced release behavior in acidic medium can be attributed to partial swelling and protonation of the chitosan-based polymer network, which facilitated diffusion of GLCD-associated active components into the surrounding medium. In contrast, reduced swelling in ethanol-containing solutions restricted molecular diffusion and consequently lowered the release rate. Such controlled release behavior may facilitate the gradual availability of antioxidant and antimicrobial functionalities during storage. However, the present release study was designed solely to evaluate release kinetics in model media and does not represent a migration or food-contact safety assessment. Establishing food-contact suitability will require comprehensive migration studies using standardized food simulants together with appropriate toxicological evaluations.

The practical produce preservation capability of the fabricated films was evaluated using fresh produce storage tests, as shown in Figure 9b. After 7 days of storage, the unwrapped control sample exhibited severe deterioration, discoloration, and microbial spoilage, whereas the samples wrapped with GLCD-incorporated films retained improved freshness and structural integrity. Among the wrapped samples, the CP3 film appeared to provide the greatest visual preservation effect, showing reduced visible deterioration compared with the control group after 7 days of storage. The GLCD-incorporated films showed reduced visible deterioration compared with the unwrapped control. These qualitative observations may be associated with the antioxidant, antibacterial, and moisture-barrier properties of the films. However, because microbial counts and quantitative physicochemical deterioration parameters were not evaluated, the present experiment does not directly demonstrate suppression of microbial growth or oxidative degradation and should be regarded as qualitative evidence of preservation performance. Additionally, the improved barrier performance likely contributed to better retention of internal moisture and reduced environmental exposure during storage. Further studies involving quantitative measurements such as weight loss, color stability, texture changes, and microbial counts are needed to confirm the observed trends.

The antibacterial activity of the fabricated films was evaluated using the agar diffusion method, and the results are presented in Figure 9c. The antibacterial activity of the fabricated films was quantitatively evaluated by measuring the inhibition-zone diameter against *E. coli*. The inhibition-zone diameters increased progressively with increasing GLCDs loading, indicating enhanced antibacterial activity. The inhibition zones were approximately 9.2 ± 0.5, 12.3 ± 0.6, 15.1 ± 0.7, and 18.0 ± 0.8 mm for CP0, CP1, CP2, and CP3, respectively. Statistical analysis showed significant differences among the film formulations (*p* < 0.05). The enhanced antibacterial performance may be related to the combined effects of the intrinsic antimicrobial activity of chitosan and the surface-active functionalities of GLCDs. The pristine polymer film exhibited relatively weak antibacterial performance, whereas the GLCD-loaded films demonstrated significantly enhanced inhibition zones against bacterial growth of *E. coli*. The antibacterial activity progressively increased with increasing GLCD concentration, with CP3 showing the largest inhibition zone among all samples. The enhanced antibacterial performance may be related to the combined antimicrobial effects of chitosan and GLCD incorporation. Chitosan is known to exhibit intrinsic antibacterial activity through interactions with negatively charged bacterial cell surfaces. Although several studies have proposed mechanisms such as membrane interaction, oxidative stress induction, and disruption of cellular functions for carbon-dot-based antimicrobial materials, these mechanisms were not directly investigated in the present study. Therefore, the observed inhibition zones only demonstrate an antibacterial phenomenon and do not establish the underlying bactericidal pathway. A schematic illustration of a hypothesized antibacterial mechanism, based on previously reported literature for carbon-dot-based antimicrobial materials, is presented in Figure 9d. Although ROS-mediated antibacterial activity has been reported for related carbon nanomaterials [51,52], direct mechanistic evidence was not investigated in the present study. Therefore, the proposed pathway is presented as an illustrative mechanism based on the observed results. The elevated ROS level subsequently causes membrane disruption, protein denaturation, and DNA damage, ultimately leading to bacterial cell death. Additionally, the nanoscale dimensions and abundant functional groups of GLCDs facilitate close interaction with microbial membranes, thereby enhancing antibacterial efficiency. The observed multifunctional behavior demonstrates the strong potential of GLCD-based nanocomposite films for sustainable active food packaging applications aimed at improving food safety and extending shelf life. The antioxidant activity observed in the DPPH [53] and ABTS [54,55,56] assays reflects the radical-scavenging capability of the films under the specific assay conditions. The relationship between antioxidant activity and antibacterial action in carbon-dot-based materials is complex and may depend on environmental conditions, surface chemistry, microbial interactions, and experimental conditions. As these mechanistic aspects were not directly investigated in the present study, the current findings do not provide direct evidence of ROS generation or ROS-mediated antibacterial activity.

Recent active-packaging studies provide useful benchmarks for evaluating the present GLCDs/chitosan/PVA films. Gao et al. reported betulin/HPβCD nanofibers exhibiting antioxidant and antibacterial activities together with strawberry-preservation performance, while Zhang et al. demonstrated quantitative antibacterial activity and evaluated strawberry preservation through *Botrytis cinerea* lesion development [13,14]. In comparison, the present system employs glycine/lipoic acid-derived carbon dots incorporated into a biodegradable chitosan/PVA matrix, providing combined antioxidant, antibacterial, optical, barrier, and film-forming functionalities. The antibacterial evaluation in the present study was limited to *E. coli*, and the produce-preservation assessment was primarily qualitative. Therefore, future studies incorporating additional microbial strains and quantitative preservation parameters, including microbial load, lesion development, weight loss, and texture changes, would provide a more comprehensive evaluation of the practical food-packaging performance of the GLCD-containing films.

### 3.7. Cytocompatibility and Cell Proliferation Study of Nanocomposite Films

**Figure 10:**
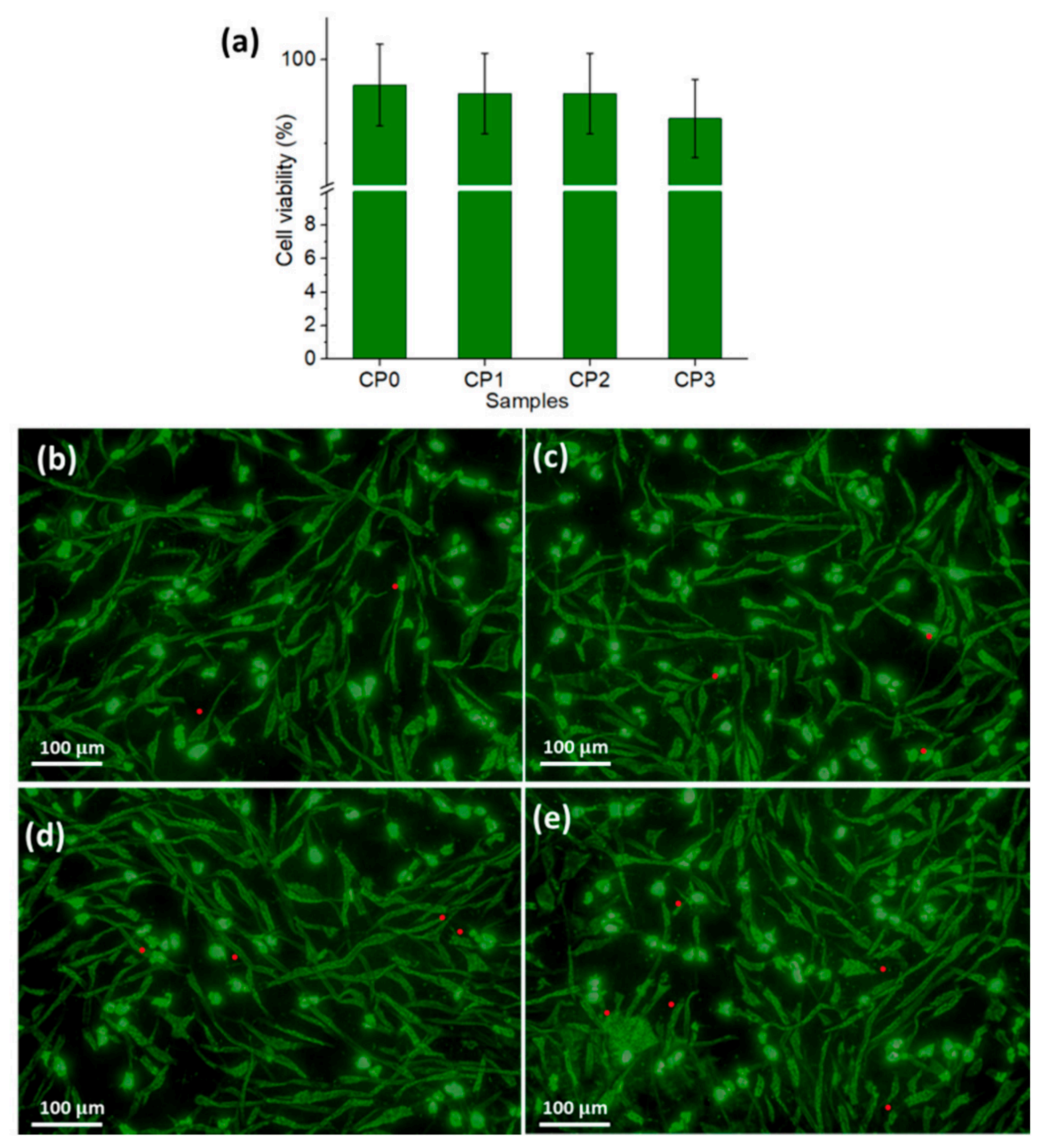
(**a**) Cell viability of NIH-3T3 fibroblast cells cultured on pristine and GLCD-incorporated chitosan/PVA nanocomposite films containing 0 wt% (CP0), 1 wt% (CP1), 3 wt% (CP2), and 5 wt% (CP3) GLCDs after 5 days of incubation. Data are presented as mean ± SD (*n* = 3). Statistical significance was determined at *p* < 0.05. (**b**–**e**) Live/dead fluorescence microscopy images of fibroblast cells cultured on CP0, CP1, CP2, and CP3 films, respectively, showing predominantly viable cells and qualitative evidence of favorable cytocompatibility. Green fluorescence represents live cells, while red fluorescence indicates dead cells. Scale bar: 100 μm.

Although the developed materials are intended for food-packaging applications, evaluation of cytocompatibility is important because active packaging systems containing nanomaterials may release trace amounts of active components during use. Therefore, NIH-3T3 fibroblast cells were employed as a preliminary in vitro model to assess the biological compatibility of the fabricated films and to determine whether GLCDs incorporation induces adverse cellular responses. The cytocompatibility results provide evidence of biocompatibility. However, food-contact safety requires additional validation through migration and toxicological studies. The cytocompatibility of the fabricated GLCD-based nanocomposite films was evaluated using NIH-3T3 fibroblast cells through a 5-day cell proliferation study, and the results are presented in Figure 10. As shown in Figure 10a, all films exhibited high cell viability values above 85%, indicating excellent biocompatibility of the developed nanocomposite system. The pristine chitosan/PVA film (CP0) showed the highest cell viability of approximately 95%, while the GLCD-incorporated films CP1 and CP2 maintained comparable viability values above 90%. Although a slight decrease in cell viability was observed for CP3, the value remained higher than 85%, confirming that the incorporation of GLCDs did not induce significant cytotoxic effects toward fibroblast cells. The high cytocompatibility of the films can be attributed to the biocompatible nature of chitosan, PVA, and the metal-free GLCDs synthesized from glycine and lipoic acid precursors. Unlike conventional metal-based nanomaterials, GLCDs possess low intrinsic toxicity and abundant surface functional groups that promote favorable interactions with cellular environments. Moreover, the favorable compatibility between GLCDs and the polymer matrix may have contributed to the observed cytocompatibility, thereby reducing potential adverse cellular responses. It should be noted that carbon-dot-based materials may potentially interfere with colorimetric assays because of their optical and fluorescent properties. Although the MTT results were supported by live/dead staining observations, specific assay-interference controls were not investigated in the present study. Therefore, the cytocompatibility results provide preliminary evidence of biological compatibility, while further validation using appropriate interference controls and complementary viability assays is necessary.

Live/dead fluorescence staining images after 5 days of culture are presented in Figure 10b–e. A high proportion of viable fibroblast cells exhibiting predominantly green fluorescence were observed on all film surfaces. The live/dead images qualitatively suggest favorable cell viability and surface colonization following culture on the films. Only a very small number of dead cells, represented by red fluorescence signals, were detected throughout the samples. The CP1 and CP2 films exhibited extensive green fluorescence with relatively few dead cells observed in the imaged regions. These observations are interpreted qualitatively, while quantitative cell-count analysis will be performed in future studies. At higher GLCD loading (CP3), the films maintained substantial cell viability and proliferation, demonstrating favorable in vitro cytocompatibility toward NIH-3T3 fibroblast cells. However, these findings provide preliminary evidence of biological compatibility, while comprehensive migration studies, testing with appropriate food simulants, oral-exposure studies, and detailed toxicological evaluations will be required in future investigations to establish the suitability of these materials for food-contact applications. The observed cellular behavior confirms that the incorporation of sulfur- and nitrogen-rich GLCDs did not compromise the biological compatibility of the chitosan/PVA matrix. Instead, the multifunctional films exhibited antibacterial and antioxidant activities while maintaining favourable fibroblast compatibility, highlighting their potential as active packaging materials. The live/dead staining results presented herein are qualitative in nature. Quantitative assessment of cell adhesion and proliferation would require image-based live/dead cell counting from multiple random microscopic fields and independently prepared samples. Such analyses were beyond the scope of the present study and will be included in future investigations.

## 4. Conclusions

In this work, multifunctional GLCD-incorporated chitosan/PVA nanocomposite films were successfully fabricated through a facile solution-casting approach for active food packaging applications. Sulfur- and nitrogen-rich carbon dots synthesized from glycine and lipoic acid precursors exhibited excellent compatibility with the chitosan/PVA polymer matrix, enabling the formation of nanocomposite films with no visible macroscopic phase separation. The incorporation of GLCDs improved the physicochemical and functional properties of the films through enhanced intermolecular interactions within the polymer network. The developed nanocomposite films demonstrated improved mechanical strength, enhanced thermal stability, reduced water vapor permeability, and superior UV-shielding performance compared with the pristine polymer film. Furthermore, the GLCD-containing films exhibited remarkable antioxidant and antibacterial activities, which progressively increased with increasing GLCD concentration. The multifunctional properties of the films showed promising preservation potential based on qualitative storage observations. Nevertheless, additional quantitative preservation studies involving physicochemical and microbiological analyses are required to fully establish their effectiveness in food-packaging applications. Importantly, the films also showed excellent cytocompatibility toward NIH-3T3 fibroblast cells with high cell viability after 5 days of culture, providing preliminary evidence of the cytocompatibility of the developed system. Nevertheless, comprehensive migration studies, food-simulant testing, oral-exposure, and detailed toxicological assessments are required before the suitability of these materials for food-contact applications can be conclusively established. The enhanced performance of the nanocomposite films can be attributed to the synergistic effects of the sulfur/nitrogen-rich GLCDs and the chitosan/PVA polymer matrix, which collectively provided strong interfacial interactions, improved barrier properties, efficient radical scavenging ability, and effective antibacterial activity. Overall, the developed GLCD-based nanocomposite films represent a promising sustainable alternative to conventional petroleum-derived packaging materials and hold strong potential for next-generation active food packaging applications aimed at improving food safety, extending shelf life, and reducing environmental impact.

## Data Availability

The raw data supporting the conclusions of this article will be made available by the authors on request.

## Supplementary Materials

The following supporting information can be downloaded at: https://www.mdpi.com/article/10.3390/polym18182215/s1, Table S1: Mechanical properties of pure chitosan, pure PVA, and GLCD-incorporated chitosan/PVA films (CP0–CP3).
