# Supplementary material for: N,S-Co-Doped Carbon Dot-Reinforced Biopolymer Films with Enhanced Barrier, Antioxidant, and Antimicrobial Performance for Active Food Packaging
Source: Polymers (Basel). 2026 Sep 11;18(18):2215. doi: 10.3390/polym18182215 (PMC13611285; doi:10.3390/polym18182215)
Supplement: Supplementary file 1 [file polymers-18-02215-s001.zip › polymers-4507640-supplementary.pdf]

Supporting Information

N,S-Co-Doped Carbon Dot-Reinforced Biopolymer Films with Enhanced Barrier, Antioxidant, and Antimicrobial Performance for Active Food Packaging

Suleiman A Althawab*

[1]Department of Food Science & Nutrition, College of Food and Agricultural Sciences, King Saud University, Riyadh 11451, Saudi Arabia

*E-mail address: Salthawab@ksu.edu.sa

Characterizations

The morphology and particle size distribution of the synthesized GLCDs were analyzed using transmission electron microscopy (TEM, JEM-2100F, JEOL Ltd., Japan). Particle diameters were measured from TEM images using ImageJ software (NIH, USA). Fourier transform infrared (FTIR) spectra were recorded using an FTIR spectrometer (Nicolet iS50, Thermo Fisher Scientific, USA) in the range of 4000–500 cm^{-1}. Ultraviolet–visible (UV–Vis) absorption spectra were obtained using a UV–Vis spectrophotometer (Cary 60, Agilent Technologies, USA). Photoluminescence (PL) measurements were carried out using a fluorescence spectrophotometer (F-7000, Hitachi, Japan). X-ray photoelectron spectroscopy (XPS) analysis was performed using an XPS system (K-Alpha+, Thermo Fisher Scientific, UK) to investigate the elemental composition and surface chemical states of the GLCDs. Mechanical properties of the films were measured using a universal testing machine (Instron 5943, Instron, USA). At least five specimens (n = 5) were tested for each film formulation, and the results are reported as mean ± standard deviation. Statistical analysis was performed using one-way analysis of variance (ANOVA), and differences were considered statistically significant at $p < 0.05$. Water contact angle measurements were conducted using a contact angle goniometer (OCA 15EC, DataPhysics Instruments GmbH, Germany). Water contact angles were measured using a sessile-drop method with a 5 µL deionized water droplet. Images were captured immediately after droplet deposition (within 5 s), and the left and right contact angles were averaged. Measurements were performed at five random positions on each film surface, and the results are reported as mean ± standard deviation. Mechanical properties were evaluated using a universal testing machine according to ASTM D882. Film specimens were cut into rectangular strips (60 mm × 10 mm) with an average thickness of 85 ± 3

μm. The initial gauge length was 30 mm, and the crosshead speed was set at 5 mm min^{-1}. Prior to testing, all films were conditioned at 25 ± 2 °C and 50 ± 5% relative humidity for at least 24 h. Five specimens were tested for each formulation (n = 5), and the results are reported as mean ± standard deviation. Thermal stability was analyzed using thermogravimetric analysis (TGA, TGA550, TA Instruments, USA) under a nitrogen atmosphere. The optical transparency of the films was evaluated using a UV-Vis spectrophotometer, while film thickness was measured using a digital micrometer (Mitutoyo Corporation, Japan). Antibacterial studies were conducted using a standard agar diffusion method, and antioxidant activity was evaluated using the DPPH free radical scavenging assay.

Antioxidant Activity Measurement

DPPH (2,2-Diphenyl-1-picrylhydrazyl) was utilized to examine the free radical scavenging capacities of the samples [35, 50, 53]. This method involves the reduction of DPPH in an alcoholic solution in the presence of a hydrogen-donating antioxidant, leading to the nonradical form of DPPH. The DPPH free radical inhibition activity was measured by monitoring the reduction in absorbance of DPPH caused by the samples. Different samples were introduced into the methanolic solution containing 1 mL of DPPH [50]. The mixtures were incubated in the dark for a specific period of time, and then the absorbance at 517 nm was measured by using Cary 60 UV-Vis Spectrophotometer. The DPPH free radical scavenging activity was assessed using the following method:

$$\% \text{ scavenging activity} = (A_c - A_s)/A_c \quad (1)$$

Where Ac and As denote the absorbance of DPPH free radicals at 517 nm in the absence and presence of the sample.

The ABTS (2,2'-azino-bis(3-ethylbenzothiazoline-6-sulfonic acid) radical cation decolorization assay was employed to assess the free radical scavenging capability[54]. 5 mL of 7 mM ABTS solution and 88 μL of 140 mM potassium persulfate solution were combined to produce the ABTS solution with a final concentration of 2.45 mM. The radical cation solution was diluted until it reached an initial absorbance value of 0.7±0.05 at 734 nm after being stored in the dark for 16 hours [55, 56]. The green dispersion was then treated with samples, and the absorbance was measured. The ABTS radical inhibition activity was measured by monitoring the decrease in absorbance of ABTS solution (using Cary 60 UV-Vis Spectrophotometer) due to the presence of the samples. This transition is evident as the color shifts from green to colorless. The equation used

to assess the antioxidant activity of ABTS was also measured according to equation (1) where A_c and A_s denote the absorbance at 734 nm in the absence and presence of the sample. All experiments were performed in triplicate.

Cytocompatibility and Cell Proliferation Assay

The cytocompatibility of the fabricated chitosan/PVA and GLCD-incorporated nanocomposite films was evaluated using NIH-3T3 mouse fibroblast cells through an MTT assay. Briefly, NIH-3T3 cells were seeded in 96-well culture plates and incubated under standard cell culture conditions. Sterilized film samples were introduced into the wells, and the cells were cultured for up to 5 days. At predetermined time intervals, cell viability was assessed using the MTT assay according to the manufacturer's protocol. After incubation with MTT reagent, the resulting formazan crystals were dissolved, and the absorbance was measured using a microplate reader. Cell viability was calculated relative to untreated control cells, which were considered as 100% viable. In addition, live/dead cell staining was performed after 5 days of culture to visualize cell adhesion, viability, and proliferation on the film surfaces. Live/dead staining was performed using Calcein-AM and propidium iodide (PI) after 5 days of culture, and the stained cells were observed using a fluorescence microscope. All experiments were conducted in triplicate (n = 3), and the results are presented as mean ± standard deviation.

Produce Preservation, and Antibacterial Evaluation

Fresh grapes were selected as a model perishable produce for storage studies. Fresh produce samples of similar size and maturity were used for storage experiments. The experimental groups included an unwrapped control (None), paraffin-wrapped samples, and samples wrapped with CP0, CP1, CP2, and CP3 films. The paraffin-treated sample was included as a reference packaging treatment for comparison with the developed biopolymer films. All samples were stored under identical environmental conditions, and their visual appearance was monitored during the storage period. Samples of similar size, maturity, and appearance were used. Individual fruits were wrapped with CP0, CP1, CP2, and CP3 films, while unwrapped fruits served as the control. Three independent samples were used for each group (n = 3). The packaged fruits were stored at ambient conditions (25 ± 2 °C and 50 ± 5% relative humidity) for 7 days. Visual appearance and spoilage characteristics were documented periodically, and representative photographs were recorded at the end of the storage period. The produce preservation performance of the fabricated films was evaluated using fresh produce storage experiments. Fresh produce samples of similar size and

maturity were individually wrapped with CP0, CP1, CP2, and CP3 films, while unwrapped produce served as the control. The samples were stored at ambient conditions for 7 days, and their visual appearance, freshness, and spoilage characteristics were recorded periodically. The preservation efficacy of the films was assessed by comparing the extent of discoloration, surface deterioration, and microbial spoilage among the different groups.

The antibacterial activity of the films was evaluated against representative Gram-negative bacteria (*E. coli*) using the agar diffusion method. Briefly, bacterial suspensions were uniformly spread onto nutrient agar plates, and circular disc samples were placed on the inoculated agar surface. The plates were incubated at 37 °C for 24 h, after which the diameter of the inhibition zone surrounding each film sample was measured. The antibacterial performance was determined by comparing the inhibition zone diameters of the pristine chitosan/PVA film and GLCDs-loaded nanocomposite films.

Table S1 Mechanical properties of pure chitosan, pure PVA, and GLCD-incorporated chitosan/PVA films (CP0-CP3)

Sample	Tensile Strength (MPa)	Elongation at Break (%)	Young's Modulus (MPa)
Pure chitosan	~4.0	~39	~25
CP0	~45.5	~117	~68
CP1	~46.0	~109	~96
CP2	~50.5	~99	~100
CP3	~52.5	~89	~98
Pure PVA	~47.5	~355	~141

References

35. Das, P., et al., *Carbon dot biopolymer-based flexible functional films for antioxidant and food monitoring applications.* ACS Applied Polymer Materials, 2022. **4**(12): p. 9323-9340.

50. Alzahrani, A., *Fluorescent carbon dots in situ polymerized biodegradable semi-interpenetrating tough hydrogel films with antioxidant and antibacterial activity for applications in food industry.* Food Chemistry, 2024. **447**: p. 138905.

53. Murugesan, B., et al., *Photoluminescent reduced graphene oxide quantum dots from latex of Calotropis gigantea for metal sensing, radical scavenging, cytotoxicity, and bioimaging in Artemia salina: A greener route.* Journal of Photochemistry and Photobiology B: Biology, 2018. **178**: p. 371-379.

54. Wojdyło, A., J. Oszmiański, and R. Czemerys, *Antioxidant activity and phenolic compounds in 32 selected herbs.* Food chemistry, 2007. **105**(3): p. 940-949.
55. Re, R., et al., *Antioxidant activity applying an improved ABTS radical cation decolorization assay.* Free radical biology and medicine, 1999. **26**(9-10): p. 1231-1237.
56. Nenadis, N., et al., *Estimation of scavenging activity of phenolic compounds using the ABTS•+ assay.* Journal of agricultural and food chemistry, 2004. **52**(15): p. 4669-4674.
